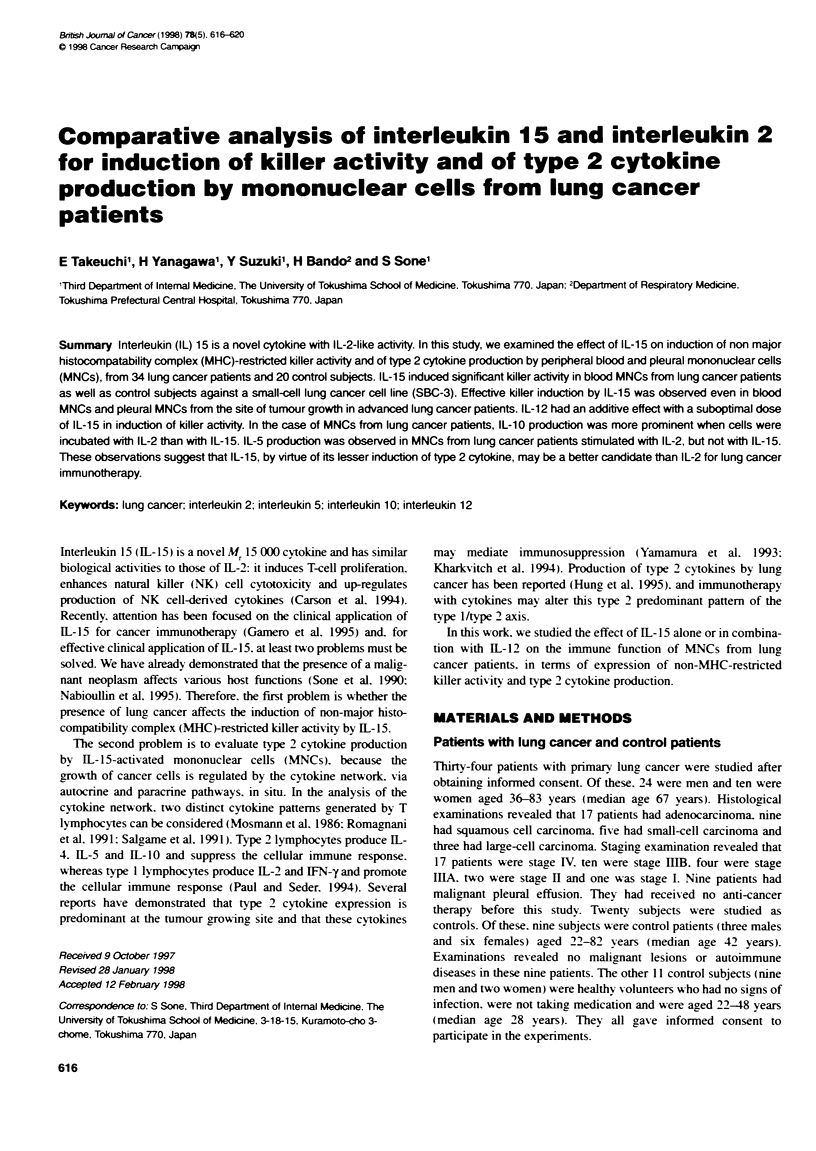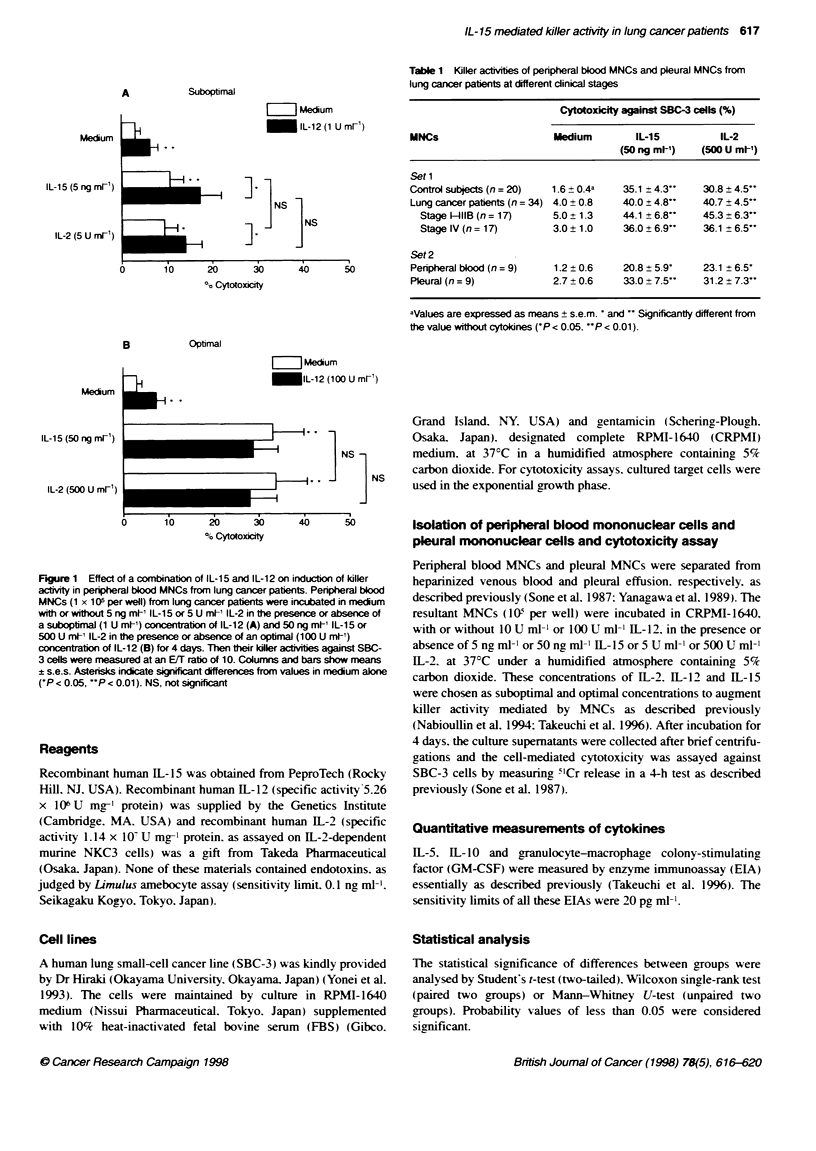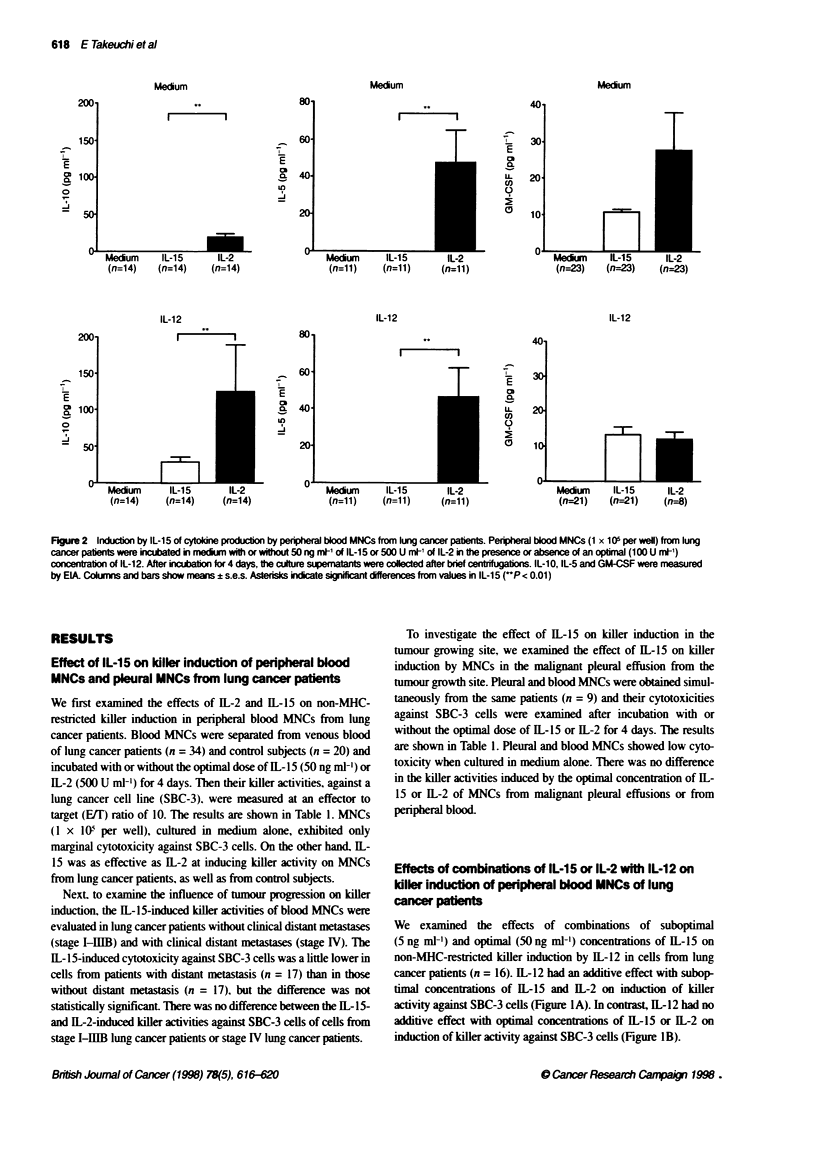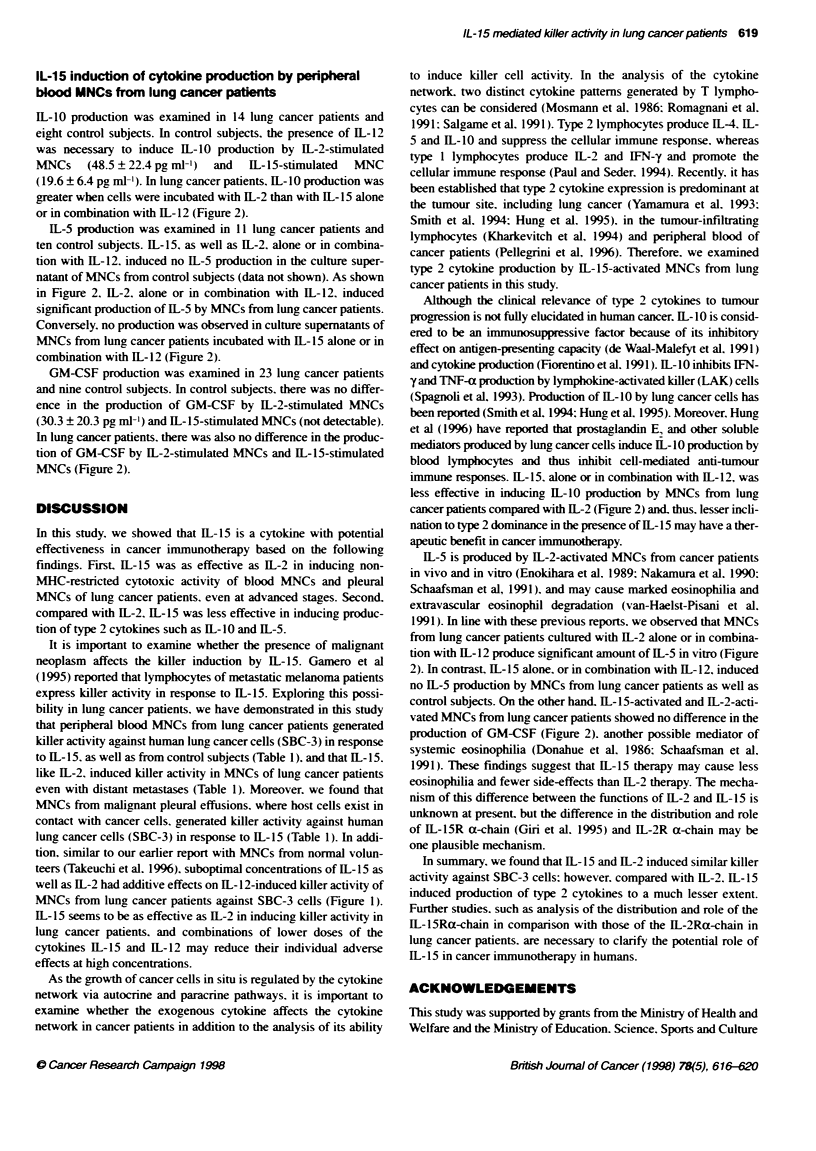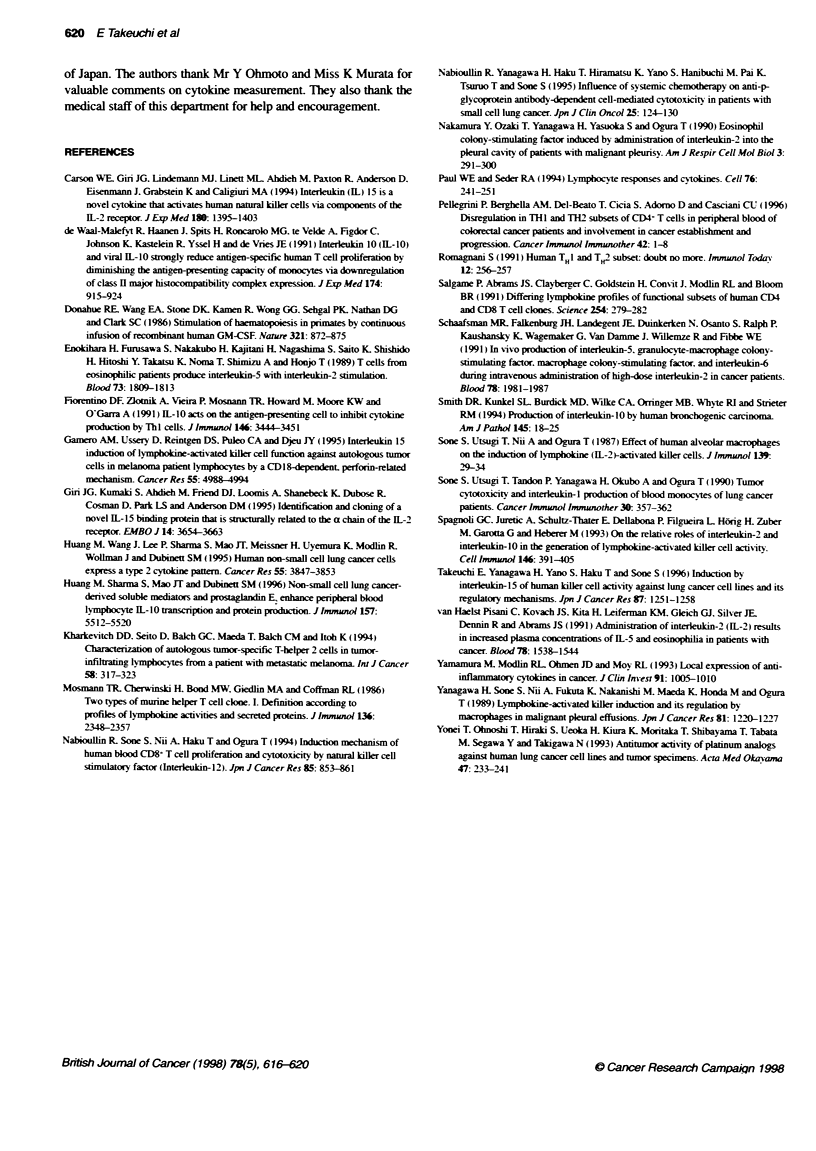# Comparative analysis of interleukin 15 and interleukin 2 for induction of killer activity and of type 2 cytokine production by mononuclear cells from lung cancer patients.

**DOI:** 10.1038/bjc.1998.550

**Published:** 1998-09

**Authors:** E. Takeuchi, H. Yanagawa, Y. Suzuki, H. Bando, S. Sone

**Affiliations:** Third Department of Internal Medicine, The University of Tokushima School of Medicine, Japan.

## Abstract

Interleukin (IL) 15 is a novel cytokine with IL-2-like activity. In this study, we examined the effect of IL-15 on induction of non major histocompatibility complex (MHC)-restricted killer activity and of type 2 cytokine production by peripheral blood and pleural mononuclear cells (MNCs), from 34 lung cancer patients and 20 control subjects. IL-15 induced significant killer activity in blood MNCs from lung cancer patients as well as control subjects against a small-cell lung cancer cell line (SBC-3). Effective killer induction by IL-15 was observed even in blood MNCs and pleural MNCs from the site of tumour growth in advanced lung cancer patients. IL-12 had an additive effect with a suboptimal dose of IL-15 in induction of killer activity. In the case of MNCs from lung cancer patients, IL-10 production was more prominent when cells were incubated with IL-2 than with IL-15. IL-5 production was observed in MNCs from lung cancer patients stimulated with IL-2, but not with IL-15. These observations suggest that IL-15, by virtue of its lesser induction of type 2 cytokine, may be a better candidate than IL-2 for lung cancer immunotherapy.


British Journal of Cancer (1998) 78(5). 616–620
© 1998 Cancer Research Campaign

### Comparative analysis of interleukin 15 and interleukin 2 for induction of killer activity and of type 2 cytokine production by mononuclear cells from lung cancer patients

**E Takeuchi[1], H Yanagawa[1], Y Suzuki[1], H Bando[2] and S Sone[1]**

[1]Third Department of Internal Medicine, The University of Tokushima School of Medicine. Tokushima 770. Japan; [2]Department of Respiratory Medicine. Tokushima Prefectural Central Hospital. Tokushima 770, Japan

**Summary** Interleukin (IL) 15 is a novel cytokine with IL-2-like activity. In this study, we examined the effect of IL-15 on induction of non major histocompatability complex (MHC)-restricted killer activity and of type 2 cytokine production by peripheral blood and pleural mononuclear cells (MNCs), from 34 lung cancer patients and 20 control subjects. IL-15 induced significant killer activity in blood MNCs from lung cancer patients as well as control subjects against a small-cell lung cancer cell line (SBC-3). Effective killer induction by IL-15 was observed even in blood MNCs and pleural MNCs from the site of tumour growth in advanced lung cancer patients. IL-12 had an additive effect with a suboptimal dose of IL-15 in induction of killer activity. In the case of MNCs from lung cancer patients, IL-10 production was more prominent when cells were incubated with IL-2 than with IL-15. IL-5 production was observed in MNCs from lung cancer patients stimulated with IL-2, but not with IL-15. These observations suggest that IL-15, by virtue of its lesser induction of type 2 cytokine, may be a better candidate than IL-2 for lung cancer immunotherapy.

**Keywords:** lung cancer; interleukin 2; interleukin 5; interleukin 10; interleukin 12

Interleukin 15 (IL-15) is a novel $M_r$ 15 000 cytokine and has similar biological activities to those of IL-2: it induces T-cell proliferation, enhances natural killer (NK) cell cytotoxicity and up-regulates production of NK cell-derived cytokines (Carson et al. 1994). Recently, attention has been focused on the clinical application of IL-15 for cancer immunotherapy (Gamero et al. 1995) and, for effective clinical application of IL-15, at least two problems must be solved. We have already demonstrated that the presence of a malignant neoplasm affects various host functions (Sone et al. 1990; Nabioullin et al. 1995). Therefore, the first problem is whether the presence of lung cancer affects the induction of non-major histocompatibility complex (MHC)-restricted killer activity by IL-15.

The second problem is to evaluate type 2 cytokine production by IL-15-activated mononuclear cells (MNCs), because the growth of cancer cells is regulated by the cytokine network, via autocrine and paracrine pathways, in situ. In the analysis of the cytokine network, two distinct cytokine patterns generated by T lymphocytes can be considered (Mosmann et al. 1986; Romagnani et al. 1991; Salgame et al. 1991). Type 2 lymphocytes produce IL-4, IL-5 and IL-10 and suppress the cellular immune response, whereas type 1 lymphocytes produce IL-2 and IFN-γ and promote the cellular immune response (Paul and Seder, 1994). Several reports have demonstrated that type 2 cytokine expression is predominant at the tumour growing site and that these cytokines may mediate immunosuppression (Yamamura et al. 1993; Kharkvitch et al. 1994). Production of type 2 cytokines by lung cancer has been reported (Hung et al. 1995), and immunotherapy with cytokines may alter this type 2 predominant pattern of the type 1/type 2 axis.

In this work, we studied the effect of IL-15 alone or in combination with IL-12 on the immune function of MNCs from lung cancer patients, in terms of expression of non-MHC-restricted killer activity and type 2 cytokine production.

*Received 9 October 1997*
*Revised 28 January 1998*
*Accepted 12 February 1998*

*Correspondence to:* S Sone, Third Department of Internal Medicine, The University of Tokushima School of Medicine, 3-18-15, Kuramoto-cho 3-chome, Tokushima 770, Japan

### MATERIALS AND METHODS

### Patients with lung cancer and control patients

Thirty-four patients with primary lung cancer were studied after obtaining informed consent. Of these, 24 were men and ten were women aged 36–83 years (median age 67 years). Histological examinations revealed that 17 patients had adenocarcinoma, nine had squamous cell carcinoma, five had small-cell carcinoma and three had large-cell carcinoma. Staging examination revealed that 17 patients were stage IV, ten were stage IIIB, four were stage IIIA, two were stage II and one was stage I. Nine patients had malignant pleural effusion. They had received no anti-cancer therapy before this study. Twenty subjects were studied as controls. Of these, nine subjects were control patients (three males and six females) aged 22–82 years (median age 42 years). Examinations revealed no malignant lesions or autoimmune diseases in these nine patients. The other 11 control subjects (nine men and two women) were healthy volunteers who had no signs of infection, were not taking medication and were aged 22–48 years (median age 28 years). They all gave informed consent to participate in the experiments.

**Figure 1** Effect of a combination of IL-15 and IL-12 on induction of killer activity in peripheral blood MNCs from lung cancer patients. Peripheral blood MNCs ($1 \times 10^5$ per well) from lung cancer patients were incubated in medium with or without 5 ng $ml^{-1}$ IL-15 or 5 U $ml^{-1}$ IL-2 in the presence or absence of a suboptimal (1 U $ml^{-1}$) concentration of IL-12 (**A**) and 50 ng $ml^{-1}$ IL-15 or 500 U $ml^{-1}$ IL-2 in the presence or absence of an optimal (100 U $ml^{-1}$) concentration of IL-12 (**B**) for 4 days. Then their killer activities against SBC-3 cells were measured at an E/T ratio of 10. Columns and bars show means ± s.e.s. Asterisks indicate significant differences from values in medium alone (*$P < 0.05$, **$P < 0.01$). NS, not significant

### Reagents

Recombinant human IL-15 was obtained from PeproTech (Rocky Hill, NJ, USA). Recombinant human IL-12 (specific activity 5.26 × $10^6$ U $mg^{-1}$ protein) was supplied by the Genetics Institute (Cambridge, MA, USA) and recombinant human IL-2 (specific activity 1.14 × $10^7$ U $mg^{-1}$ protein, as assayed on IL-2-dependent murine NKC3 cells) was a gift from Takeda Pharmaceutical (Osaka, Japan). None of these materials contained endotoxins, as judged by *Limulus* amebocyte assay (sensitivity limit, 0.1 ng $ml^{-1}$, Seikagaku Kogyo, Tokyo, Japan).

### Cell lines

A human lung small-cell cancer line (SBC-3) was kindly provided by Dr Hiraki (Okayama University, Okayama, Japan) (Yonei et al, 1993). The cells were maintained by culture in RPMI-1640 medium (Nissui Pharmaceutical, Tokyo, Japan) supplemented with 10% heat-inactivated fetal bovine serum (FBS) (Gibco, Grand Island, NY, USA) and gentamicin (Schering-Plough, Osaka, Japan), designated complete RPMI-1640 (CRPMI) medium, at 37°C in a humidified atmosphere containing 5% carbon dioxide. For cytotoxicity assays, cultured target cells were used in the exponential growth phase.

**Table 1** Killer activities of peripheral blood MNCs and pleural MNCs from lung cancer patients at different clinical stages

| MNCs | Cytotoxicity against SBC-3 cells (%) | | |
|---|---|---|---|
| | **Medium** | **IL-15 (50 ng ml⁻¹)** | **IL-2 (500 U ml⁻¹)** |
| *Set* 1 | | | |
| Control subjects (*n* = 20) | 1.6 ± 0.4[a] | 35.1 ± 4.3** | 30.8 ± 4.5** |
| Lung cancer patients (*n* = 34) | 4.0 ± 0.8 | 40.0 ± 4.8** | 40.7 ± 4.5** |
| Stage I–IIIB (*n* = 17) | 5.0 ± 1.3 | 44.1 ± 6.8** | 45.3 ± 6.3** |
| Stage IV (*n* = 17) | 3.0 ± 1.0 | 36.0 ± 6.9** | 36.1 ± 6.5** |
| *Set* 2 | | | |
| Peripheral blood (*n* = 9) | 1.2 ± 0.6 | 20.8 ± 5.9* | 23.1 ± 6.5* |
| Pleural (*n* = 9) | 2.7 ± 0.6 | 33.0 ± 7.5** | 31.2 ± 7.3** |

[a]Values are expressed as means ± s.e.m. * and ** Significantly different from the value without cytokines (*$P < 0.05$, **$P < 0.01$).

### Isolation of peripheral blood mononuclear cells and pleural mononuclear cells and cytotoxicity assay

Peripheral blood MNCs and pleural MNCs were separated from heparinized venous blood and pleural effusion, respectively, as described previously (Sone et al, 1987; Yanagawa et al, 1989). The resultant MNCs ($10^5$ per well) were incubated in CRPMI-1640, with or without 10 U $ml^{-1}$ or 100 U $ml^{-1}$ IL-12, in the presence or absence of 5 ng $ml^{-1}$ or 50 ng $ml^{-1}$ IL-15 or 5 U $ml^{-1}$ or 500 U $ml^{-1}$ IL-2, at 37°C under a humidified atmosphere containing 5% carbon dioxide. These concentrations of IL-2, IL-12 and IL-15 were chosen as suboptimal and optimal concentrations to augment killer activity mediated by MNCs as described previously (Nabioullin et al, 1994; Takeuchi et al, 1996). After incubation for 4 days, the culture supernatants were collected after brief centrifugations and the cell-mediated cytotoxicity was assayed against SBC-3 cells by measuring $^{51}Cr$ release in a 4-h test as described previously (Sone et al, 1987).

### Quantitative measurements of cytokines

IL-5, IL-10 and granulocyte–macrophage colony-stimulating factor (GM-CSF) were measured by enzyme immunoassay (EIA) essentially as described previously (Takeuchi et al, 1996). The sensitivity limits of all these EIAs were 20 pg $ml^{-1}$.

### Statistical analysis

The statistical significance of differences between groups were analysed by Student's *t*-test (two-tailed), Wilcoxon single-rank test (paired two groups) or Mann–Whitney *U*-test (unpaired two groups). Probability values of less than 0.05 were considered significant.

© Cancer Research Campaign 1998

**Figure 2** Induction by IL-15 of cytokine production by peripheral blood MNCs from lung cancer patients. Peripheral blood MNCs (1 × $10^5$ per well) from lung cancer patients were incubated in medium with or without 50 ng ml$^{-1}$ of IL-15 or 500 U ml$^{-1}$ of IL-2 in the presence or absence of an optimal (100 U ml$^{-1}$) concentration of IL-12. After incubation for 4 days, the culture supernatants were collected after brief centrifugations. IL-10, IL-5 and GM-CSF were measured by EIA. Columns and bars show means ± s.e.s. Asterisks indicate significant differences from values in IL-15 (**$P < 0.01$)

### RESULTS

### Effect of IL-15 on killer induction of peripheral blood MNCs and pleural MNCs from lung cancer patients

We first examined the effects of IL-2 and IL-15 on non-MHC-restricted killer induction in peripheral blood MNCs from lung cancer patients. Blood MNCs were separated from venous blood of lung cancer patients ($n$ = 34) and control subjects ($n$ = 20) and incubated with or without the optimal dose of IL-15 (50 ng ml$^{-1}$) or IL-2 (500 U ml$^{-1}$) for 4 days. Then their killer activities, against a lung cancer cell line (SBC-3), were measured at an effector to target (E/T) ratio of 10. The results are shown in Table 1. MNCs (1 × $10^5$ per well), cultured in medium alone, exhibited only marginal cytotoxicity against SBC-3 cells. On the other hand, IL-15 was as effective as IL-2 at inducing killer activity on MNCs from lung cancer patients, as well as from control subjects.

Next, to examine the influence of tumour progression on killer induction, the IL-15-induced killer activities of blood MNCs were evaluated in lung cancer patients without clinical distant metastases (stage I–IIIB) and with clinical distant metastases (stage IV). The IL-15-induced cytotoxicity against SBC-3 cells was a little lower in cells from patients with distant metastasis ($n$ = 17) than in those without distant metastasis ($n$ = 17), but the difference was not statistically significant. There was no difference between the IL-15- and IL-2-induced killer activities against SBC-3 cells of cells from stage I–IIIB lung cancer patients or stage IV lung cancer patients.

To investigate the effect of IL-15 on killer induction in the tumour growing site, we examined the effect of IL-15 on killer induction by MNCs in the malignant pleural effusion from the tumour growth site. Pleural and blood MNCs were obtained simultaneously from the same patients ($n$ = 9) and their cytotoxicities against SBC-3 cells were examined after incubation with or without the optimal dose of IL-15 or IL-2 for 4 days. The results are shown in Table 1. Pleural and blood MNCs showed low cytotoxicity when cultured in medium alone. There was no difference in the killer activities induced by the optimal concentration of IL-15 or IL-2 of MNCs from malignant pleural effusions or from peripheral blood.

### Effects of combinations of IL-15 or IL-2 with IL-12 on killer induction of peripheral blood MNCs of lung cancer patients

We examined the effects of combinations of suboptimal (5 ng ml$^{-1}$) and optimal (50 ng ml$^{-1}$) concentrations of IL-15 on non-MHC-restricted killer induction by IL-12 in cells from lung cancer patients ($n$ = 16). IL-12 had an additive effect with suboptimal concentrations of IL-15 and IL-2 on induction of killer activity against SBC-3 cells (Figure 1A). In contrast, IL-12 had no additive effect with optimal concentrations of IL-15 or IL-2 on induction of killer activity against SBC-3 cells (Figure 1B).

© Cancer Research Campaign 1998

### IL-15 induction of cytokine production by peripheral blood MNCs from lung cancer patients

IL-10 production was examined in 14 lung cancer patients and eight control subjects. In control subjects, the presence of IL-12 was necessary to induce IL-10 production by IL-2-stimulated MNCs ($48.5 \pm 22.4$ pg ml$^{-1}$) and IL-15-stimulated MNC ($19.6 \pm 6.4$ pg ml$^{-1}$). In lung cancer patients, IL-10 production was greater when cells were incubated with IL-2 than with IL-15 alone or in combination with IL-12 (Figure 2).

IL-5 production was examined in 11 lung cancer patients and ten control subjects. IL-15, as well as IL-2, alone or in combination with IL-12, induced no IL-5 production in the culture supernatant of MNCs from control subjects (data not shown). As shown in Figure 2, IL-2, alone or in combination with IL-12, induced significant production of IL-5 by MNCs from lung cancer patients. Conversely, no production was observed in culture supernatants of MNCs from lung cancer patients incubated with IL-15 alone or in combination with IL-12 (Figure 2).

GM-CSF production was examined in 23 lung cancer patients and nine control subjects. In control subjects, there was no difference in the production of GM-CSF by IL-2-stimulated MNCs ($30.3 \pm 20.3$ pg ml$^{-1}$) and IL-15-stimulated MNCs (not detectable). In lung cancer patients, there was also no difference in the production of GM-CSF by IL-2-stimulated MNCs and IL-15-stimulated MNCs (Figure 2).

### DISCUSSION

In this study, we showed that IL-15 is a cytokine with potential effectiveness in cancer immunotherapy based on the following findings. First, IL-15 was as effective as IL-2 in inducing non-MHC-restricted cytotoxic activity of blood MNCs and pleural MNCs of lung cancer patients, even at advanced stages. Second, compared with IL-2, IL-15 was less effective in inducing production of type 2 cytokines such as IL-10 and IL-5.

It is important to examine whether the presence of malignant neoplasm affects the killer induction by IL-15. Gamero et al (1995) reported that lymphocytes of metastatic melanoma patients express killer activity in response to IL-15. Exploring this possibility in lung cancer patients, we have demonstrated in this study that peripheral blood MNCs from lung cancer patients generated killer activity against human lung cancer cells (SBC-3) in response to IL-15, as well as from control subjects (Table 1), and that IL-15, like IL-2, induced killer activity in MNCs of lung cancer patients even with distant metastases (Table 1). Moreover, we found that MNCs from malignant pleural effusions, where host cells exist in contact with cancer cells, generated killer activity against human lung cancer cells (SBC-3) in response to IL-15 (Table 1). In addition, similar to our earlier report with MNCs from normal volunteers (Takeuchi et al, 1996), suboptimal concentrations of IL-15 as well as IL-2 had additive effects on IL-12-induced killer activity of MNCs from lung cancer patients against SBC-3 cells (Figure 1). IL-15 seems to be as effective as IL-2 in inducing killer activity in lung cancer patients, and combinations of lower doses of the cytokines IL-15 and IL-12 may reduce their individual adverse effects at high concentrations.

As the growth of cancer cells in situ is regulated by the cytokine network via autocrine and paracrine pathways, it is important to examine whether the exogenous cytokine affects the cytokine network in cancer patients in addition to the analysis of its ability to induce killer cell activity. In the analysis of the cytokine network, two distinct cytokine patterns generated by T lymphocytes can be considered (Mosmann et al, 1986; Romagnani et al, 1991; Salgame et al, 1991). Type 2 lymphocytes produce IL-4, IL-5 and IL-10 and suppress the cellular immune response, whereas type 1 lymphocytes produce IL-2 and IFN-$\gamma$ and promote the cellular immune response (Paul and Seder, 1994). Recently, it has been established that type 2 cytokine expression is predominant at the tumour site, including lung cancer (Yamamura et al, 1993; Smith et al, 1994; Hung et al, 1995), in the tumour-infiltrating lymphocytes (Kharkevitch et al, 1994) and peripheral blood of cancer patients (Pellegrini et al, 1996). Therefore, we examined type 2 cytokine production by IL-15-activated MNCs from lung cancer patients in this study.

Although the clinical relevance of type 2 cytokines to tumour progression is not fully elucidated in human cancer, IL-10 is considered to be an immunosuppressive factor because of its inhibitory effect on antigen-presenting capacity (de Waal-Malefyt et al, 1991) and cytokine production (Fiorentino et al, 1991). IL-10 inhibits IFN-$\gamma$ and TNF-$\alpha$ production by lymphokine-activated killer (LAK) cells (Spagnoli et al, 1993). Production of IL-10 by lung cancer cells has been reported (Smith et al, 1994; Hung et al, 1995). Moreover, Hung et al (1996) have reported that prostaglandin $E_2$ and other soluble mediators produced by lung cancer cells induce IL-10 production by blood lymphocytes and thus inhibit cell-mediated anti-tumour immune responses. IL-15, alone or in combination with IL-12, was less effective in inducing IL-10 production by MNCs from lung cancer patients compared with IL-2 (Figure 2) and, thus, lesser inclination to type 2 dominance in the presence of IL-15 may have a therapeutic benefit in cancer immunotherapy.

IL-5 is produced by IL-2-activated MNCs from cancer patients in vivo and in vitro (Enokihara et al, 1989; Nakamura et al, 1990; Schaafsman et al, 1991), and may cause marked eosinophilia and extravascular eosinophil degradation (van-Haelst-Pisani et al, 1991). In line with these previous reports, we observed that MNCs from lung cancer patients cultured with IL-2 alone or in combination with IL-12 produce significant amount of IL-5 in vitro (Figure 2). In contrast, IL-15 alone, or in combination with IL-12, induced no IL-5 production by MNCs from lung cancer patients as well as control subjects. On the other hand, IL-15-activated and IL-2-activated MNCs from lung cancer patients showed no difference in the production of GM-CSF (Figure 2), another possible mediator of systemic eosinophilia (Donahue et al, 1986; Schaafsman et al, 1991). These findings suggest that IL-15 therapy may cause less eosinophilia and fewer side-effects than IL-2 therapy. The mechanism of this difference between the functions of IL-2 and IL-15 is unknown at present, but the difference in the distribution and role of IL-15R $\alpha$-chain (Giri et al, 1995) and IL-2R $\alpha$-chain may be one plausible mechanism.

In summary, we found that IL-15 and IL-2 induced similar killer activity against SBC-3 cells; however, compared with IL-2, IL-15 induced production of type 2 cytokines to a much lesser extent. Further studies, such as analysis of the distribution and role of the IL-15R$\alpha$-chain in comparison with those of the IL-2R$\alpha$-chain in lung cancer patients, are necessary to clarify the potential role of IL-15 in cancer immunotherapy in humans.

### ACKNOWLEDGEMENTS

This study was supported by grants from the Ministry of Health and Welfare and the Ministry of Education, Science, Sports and Culture

© Cancer Research Campaign 1998

of Japan. The authors thank Mr Y Ohmoto and Miss K Murata for valuable comments on cytokine measurement. They also thank the medical staff of this department for help and encouragement.

### REFERENCES

Carson WE. Giri JG. Lindemann MJ. Linett ML. Ahdieh M. Paxton R. Anderson D. Eisenmann J. Grabstein K and Caligiuri MA (1994) Interleukin (IL) 15 is a novel cytokine that activates human natural killer cells via components of the IL-2 receptor. *J Exp Med* **180**: 1395–1403

de Waal-Malefyt R. Haanen J. Spits H. Roncarolo MG. te Velde A. Figdor C. Johnson K. Kastelein R. Yssel H and de Vries JE (1991) Interleukin 10 (IL-10) and viral IL-10 strongly reduce antigen-specific human T cell proliferation by diminishing the antigen-presenting capacity of monocytes via downregulation of class II major histocompatibility complex expression. *J Exp Med* **174**: 915–924

Donahue RE. Wang EA. Stone DK. Kamen R. Wong GG. Sehgal PK. Nathan DG and Clark SC (1986) Stimulation of haematopoiesis in primates by continuous infusion of recombinant human GM-CSF. *Nature* **321**: 872–875

Enokihara H. Furusawa S. Nakakubo H. Kajitani H. Nagashima S. Saito K. Shishido H. Hitoshi Y. Takatsu K. Noma T. Shimizu A and Honjo T (1989) T cells from eosinophilic patients produce interleukin-5 with interleukin-2 stimulation. *Blood* **73**: 1809–1813

Fiorentino DF. Zlotnik A. Vieira P. Mosmann TR. Howard M. Moore KW and O'Garra A (1991) IL-10 acts on the antigen-presenting cell to inhibit cytokine production by Th1 cells. *J Immunol* **146**: 3444–3451

Gamero AM. Ussery D. Reintgen DS. Puleo CA and Djeu JY (1995) Interleukin 15 induction of lymphokine-activated killer cell function against autologous tumor cells in melanoma patient lymphocytes by a CD18-dependent, perforin-related mechanism. *Cancer Res* **55**: 4988–4994

Giri JG. Kumaki S. Ahdieh M. Friend DJ. Loomis A. Shanebeck K. Dubose R. Cosman D. Park LS and Anderson DM (1995) Identification and cloning of a novel IL-15 binding protein that is structurally related to the α chain of the IL-2 receptor. *EMBO J* **14**: 3654–3663

Huang M. Wang J. Lee P. Sharma S. Mao JT. Meissner H. Uyemura K. Modlin R. Wollman J and Dubinett SM (1995) Human non-small cell lung cancer cells express a type 2 cytokine pattern. *Cancer Res* **55**: 3847–3853

Huang M. Sharma S. Mao JT and Dubinett SM (1996) Non-small cell lung cancer-derived soluble mediators and prostaglandin $E_2$ enhance peripheral blood lymphocyte IL-10 transcription and protein production. *J Immunol* **157**: 5512–5520

Kharkevitch DD. Seito D. Balch GC. Maeda T. Balch CM and Itoh K (1994) Characterization of autologous tumor-specific T-helper 2 cells in tumor-infiltrating lymphocytes from a patient with metastatic melanoma. *Int J Cancer* **58**: 317–323

Mosmann TR. Cherwinski H. Bond MW. Giedlin MA and Coffman RL (1986) Two types of murine helper T cell clone. I. Definition according to profiles of lymphokine activities and secreted proteins. *J Immunol* **136**: 2348–2357

Nabioullin R. Sone S. Nii A. Haku T and Ogura T (1994) Induction mechanism of human blood CD8+ T cell proliferation and cytotoxicity by natural killer cell stimulatory factor (Interleukin-12). *Jpn J Cancer Res* **85**: 853–861

Nabioullin R. Yanagawa H. Haku T. Hiramatsu K. Yano S. Hanibuchi M. Pai K. Tsuruo T and Sone S (1995) Influence of systemic chemotherapy on anti-p-glycoprotein antibody-dependent cell-mediated cytotoxicity in patients with small cell lung cancer. *Jpn J Clin Oncol* **25**: 124–130

Nakamura Y. Ozaki T. Yanagawa H. Yasuoka S and Ogura T (1990) Eosinophil colony-stimulating factor induced by administration of interleukin-2 into the pleural cavity of patients with malignant pleurisy. *Am J Respir Cell Mol Biol* **3**: 291–300

Paul WE and Seder RA (1994) Lymphocyte responses and cytokines. *Cell* **76**: 241–251

Pellegrini P. Berghella AM. Del-Beato T. Cicia S. Adorno D and Casciani CU (1996) Disregulation in TH1 and TH2 subsets of CD4+ T cells in peripheral blood of colorectal cancer patients and involvement in cancer establishment and progression. *Cancer Immunol Immunother* **42**: 1–8

Romagnani S (1991) Human $T_H1$ and $T_H2$ subset: doubt no more. *Immunol Today* **12**: 256–257

Salgame P. Abrams JS. Clayberger C. Goldstein H. Convit J. Modlin RL and Bloom BR (1991) Differing lymphokine profiles of functional subsets of human CD4 and CD8 T cell clones. *Science* **254**: 279–282

Schaafsman MR. Falkenburg JH. Landegent JE. Duinkerken N. Osanto S. Ralph P. Kaushansky K. Wagemaker G. Van Damme J. Willemze R and Fibbe WE (1991) In vivo production of interleukin-5, granulocyte-macrophage colony-stimulating factor, macrophage colony-stimulating factor, and interleukin-6 during intravenous administration of high-dose interleukin-2 in cancer patients. *Blood* **78**: 1981–1987

Smith DR. Kunkel SL. Burdick MD. Wilke CA. Orringer MB. Whyte RI and Strieter RM (1994) Production of interleukin-10 by human bronchogenic carcinoma. *Am J Pathol* **145**: 18–25

Sone S. Utsugi T. Nii A and Ogura T (1987) Effect of human alveolar macrophages on the induction of lymphokine (IL-2)-activated killer cells. *J Immunol* **139**: 29–34

Sone S. Utsugi T. Tandon P. Yanagawa H. Okubo A and Ogura T (1990) Tumor cytotoxicity and interleukin-1 production of blood monocytes of lung cancer patients. *Cancer Immunol Immunother* **30**: 357–362

Spagnoli GC. Juretic A. Schultz-Thater E. Dellabona P. Filgueira L. Hörig H. Zuber M. Garotta G and Heberer M (1993) On the relative roles of interleukin-2 and interleukin-10 in the generation of lymphokine-activated killer cell activity. *Cell Immunol* **146**: 391–405

Takeuchi E. Yanagawa H. Yano S. Haku T and Sone S (1996) Induction by interleukin-15 of human killer cell activity against lung cancer cell lines and its regulatory mechanisms. *Jpn J Cancer Res* **87**: 1251–1258

van Haelst Pisani C. Kovach JS. Kita H. Leiferman KM. Gleich GJ. Silver JE. Dennin R and Abrams JS (1991) Administration of interleukin-2 (IL-2) results in increased plasma concentrations of IL-5 and eosinophilia in patients with cancer. *Blood* **78**: 1538–1544

Yamamura M. Modlin RL. Ohmen JD and Moy RL (1993) Local expression of anti-inflammatory cytokines in cancer. *J Clin Invest* **91**: 1005–1010

Yanagawa H. Sone S. Nii A. Fukuta K. Nakanishi M. Maeda K. Honda M and Ogura T (1989) Lymphokine-activated killer induction and its regulation by macrophages in malignant pleural effusions. *Jpn J Cancer Res* **81**: 1220–1227

Yonei T. Ohnoshi T. Hiraki S. Ueoka H. Kiura K. Moritaka T. Shibayama T. Tabata M. Segawa Y and Takigawa N (1993) Antitumor activity of platinum analogs against human lung cancer cell lines and tumor specimens. *Acta Med Okayama* **47**: 233–241

© Cancer Research Campaign 1998